# A *mcr-1*-Carrying Conjugative IncX4 Plasmid in Colistin-Resistant *Escherichia coli* ST278 Strain Isolated From Dairy Cow Feces in Shanghai, China

**DOI:** 10.3389/fmicb.2018.02833

**Published:** 2018-11-30

**Authors:** Fengjia Bai, Xiaobin Li, Ben Niu, Zhaohuan Zhang, Pradeep K. Malakar, Haiquan Liu, Yingjie Pan, Yong Zhao

**Affiliations:** ^1^College of Food Science and Technology, Shanghai Ocean University, Shanghai, China; ^2^State Key Laboratory of Microbial Metabolism, Joint International Laboratory of Metabolic and Developmental Sciences, School of Life Sciences and Biotechnology, Shanghai Jiao Tong University, Shanghai, China; ^3^Laboratory of Quality & Safety Risk Assessment for Aquatic Products on Storage and Preservation (Shanghai), Ministry of Agriculture, Shanghai, China; ^4^Engineering Research Center of Food Thermal-processing Technology, Shanghai Ocean University, Shanghai, China; ^5^Shanghai Engineering Research Center of Aquatic-Product Processing & Preservation, Shanghai, China

**Keywords:** colistin resistance, *mcr-1*, *Escherichia coli*, IncX4 plasmid, whole genome sequence

## Abstract

*Enterobacteriaceae*, including *Escherichia coli*, has been shown to acquire the colistin resistance gene *mcr-1*. A strain of *E. coli*, EC11, which is resistant to colistin, polymyxin B and trimethoprim-sulfamethoxazole, was isolated in 2016 from the feces of a dairy cow in Shanghai, China. Strain EC11 identifies with sequence type ST278 and is susceptible to 19 frequently used antibiotics. Whole genome sequencing of strain EC11 showed that this strain contains a 31-kb resistance plasmid, pEC11b, which belongs to the IncX4 group. The *mcr-1* gene was shown to be inserted into a 2.6-kb *mcr-1*-*pap2* cassette of pEC11b. Plasmid pEC11b also contained putative conjugal transfer components, including an *oriT*-like region, relaxase, type IV coupling protein, and type IV secretion system. We were successful in transferring pEC11b to *E. coli* C600 with an average transconjugation efficiency of 4.6 × 10^-5^. Additionally, a MLST-based analysis comparing EC11 and other reported *mcr*-positive *E. coli* populations showed high genotypic diversity. The discovery of the *E. coli* strain EC11 with resistance to colistin in Shanghai emphasizes the importance of vigilance in detecting new threats like *mcr* genes to public health. Detection of *mcr* genes helps in tracking, slowing, and responding to the emergence of antibiotic resistance in Chinese livestock farming.

## Introduction

Antimicrobial resistance is becoming a great challenge to public health worldwide (Laxminarayan et al., 2014). The rapid evolution of MDR Gram-negative bacteria is pushing humankind to the cusp of a post-antibiotic era. Colistin (polymyxins E) is a family of cationic polypeptide antibiotics which acts as the last line of defense in the treatment of severe bacterial infections by MDR or XDR bacteria. In particular, colistin is used to treat ESBL-producing and CRE infections (Li et al., 2006; Paterson and Harris, 2016).

Colistin resistance was assumed to be chromosomally mediated, non-transmissible and an intrinsic property of the bacteria (Olaitan et al., 2014). However, the recent discovery of the *Escherichia coli* harboring plasmid-borne colistin resistance gene *mcr-1* confirms transmission of colistin resistance by HGT (Liu et al., 2016). The MCR-1 encodes a PEA transferase that adds PEA to the lipid A of the lipopolysaccharide, leading to Gram-negative bacteria resistant to colistin (Anandan et al., 2017). This HGT mechanism of colistin resistance has alarmed the medical, media, academic and public health communities.

The global spread of the *mcr-1* gene is now evident and being documented. Currently, researchers have discovered five *mcr*-like genes, ranging from *mcr-1* to *mcr-5*, with a series of *mcr* genetic variants such as *mcr-1.2, mcr-1.3* …*mcr-1.12.* These *mcr* genes have spread to 40 countries across 5 of 7 continents in multiple ecosystems, including the environment, food, animals (e.g., pig, poultry, and cattle) and humans, and in over 11 species of Enterobacteriaceae (Schwarz and Johnson, 2016; Chen et al., 2017; Feng, 2018). Retrospective studies have shown that an isolate harboring the *mcr-1* gene had already existed in three chicken *E. coli* isolates in China from the 1980s (Shen et al., 2016). The presence of *mcr-1* in livestock is indicative of the route of *mcr-1* dissemination through the food chain and it is gravely concerning that animal-to-human transmission of MCR-1 colistin resistance has already been found in many countries.

Mobile genetic elements such as conjugative plasmids, transposons, integrons and IS are important vehicles of HGT of the *mcr-1* gene (Frost et al., 2005; Sun et al., 2018). Conjugative plasmids are the main driving force for the dissemination of the *mcr-1* gene, and the plasmids IncI2 and IncX4 are the two leading plasmid types for facilitating the global dissemination of colistin resistance (Matamoros et al., 2017; Wang et al., 2018). The *mcr-1* gene is part of an approximately 2.6-kb *mcr-1-pap2* element that contains the likely promoter regions for *mcr-1* transcription (Poirel et al., 2016; Wang et al., 2018). There are also rare cases involving chromosomally integrated *mcr-1*genes (Veldman et al., 2016; Tada et al., 2017), which are indicative of non-lineage-specific vertical dissemination of *mcr-1.*

Detection of *mcr* genes helps in the tracking, slowing, and responding to the emergence of antibiotic resistance in Chinese livestock farming. At the end of 2015, the *mcr-1-*harboring *E. coli* strain SHP45 was isolated from pigs in Shanghai (Liu et al., 2016). Also, in 2016, the colistin-resistant *E. coli* EC11 strain was isolated from cow feces collected from a commercial dairy farm. We will use WGS to outline the mechanism for acquiring and transferring colistin resistance in this strain.

## Materials and Methods

### Bacterial Strains and Identification

In May 2016, we cultured *E. coli* strains from fecal samples collected from a commercial dairy farm in Shanghai, China. Samples (25 g) were dispensed in sterile plastic bags containing 225 ml of Mueller–Hinton broth and incubated at 37°C for 24 h. All samples were seeded on MacConkey agar plates with 2 μg/mL colistin and incubated at 37°C for 18 h. One putative positive *E. coli* colony per sample was selected on the basis of morphology, size, and color (peachblow), then inoculated overnight on eosin-methylene blue agar. Species were further confirmed by the amplification and sequencing of 16S rRNA, while SEM and TEM image analyses were conducted. All bacterial isolates were stored in the Luria-Bertani medium (Land Bridge, Beijing, China) with 30% glycerol at -80°C.

### *mcr-1* and β-Lactamase Gene Screening

Screening for the *mcr-1* gene was performed using PCR amplification and sequencing. The specific primers used to produce the 309 bp amplicon were as previously described: CLR5-F (5′-CGGTCAGTCCGTTTGTTC-3′) and CLR5-R (5′-CTTGGTCGGTCTGTAGGG-3′) (Liu et al., 2016). Further screening for the presence of the *mcr-2, mcr-3* and the main β-lactamase gene groups (*bla*_TEM_, *bla*_SHV_, *bla*_CTX-M_, *bla*_KPC_, and *bla*_NDM_) was performed by previously reported primers. In this study, all primers used are presented in Supplementary Table S1. Each PCR reaction system was performed in 25 μL, containing 12.5 μL of PCR Mix (Sangon Biotech, Shanghai, China), 9.5 μL of dd H_2_O, 1 μL of forward and reverse primers, and 1 μL of DNA template. Finally, one *E. coli* isolate designated as *E. coli* EC11 was determined to harbor the *mcr-1* gene, and this isolate was selected to perform the follow-up experiments.

### Antibiotic Susceptibility Testing

The MIC for 22 common antibiotics was determined for the isolate of *E. coli* EC11 by the broth dilution method on Mueller–Hinton broth (Oxoid, United Kingdom) following incubation at 37°C for 18–24 h. In this study, the 22 tested antibiotics we used are categorized into seven groups as shown in Table 1. The results were interpreted according to CLSI document M100-S25 (2015)^1^ except for tigecycline and colistin, which were interpreted by the EUCAST (version 6.0)^2^ guidelines. The double disk test (ceftazidime + ceftazidime/clavulanic acid and cefotaxime + cefotaxime/clavulanic acid) was performed to confirm the ESBL phenotype, and *E. coli* ATCC 25922 was used as a quality control.

**Table 1:** Minimum inhibitory concentration (μg/mL) for *Escherichia coli* EC11, transconjugant EC11-T and recipient *E. coli* C600.

Type of antibiotic	Antibiotic	MIC (μg/mL)^∗^		
		Donor	Transconjugant	Recipient
		*E. coli* EC11	E11-T	*E. coli* C600
β-lactams	Amoxicillin-clavulanic	2(S)	2(S)	2(S)
	Ampicillin	4(S)	8(S)	8(S)
	Piperacillin	2(S)	4(S)	4(S)
	Cefotaxime	<0.125(S)	0.25(S)	0.25(S)
	Ceftazidime	0.25(S)	0.5(S)	1(S)
	Cefoxitin	8(S)	4(S)	4(S)
	Cephazolin	2(S)	4(S)	4(S)
	Cefepime	<0.125(S)	0.25(S)	<0.125(S)
	Imipenem	0.5(S)	1(S)	0.5(S)
	Meropenem	<0.125(S)	<0.125(S)	<0.125(S)
Aminoglycoside	Amikacin	4(S)	4(S)	8(S)
	Gentamicin	2(S)	1(S)	1(S)
	Kanamycin	4(S)	4(S)	4(S)
Tetracycline	Tetracycline	1(S)	1(S)	1(S)
	Tigecycline	<0.125(S)	<0.125(S)	<0.125(S)
Quinolone	Ciprofloxacin	<0.125(S)	0.125(S)	<0.125(S)
	Levofloxacin	<0.125(S)	0.25(S)	0.5(S)
	Nalidixic acid	4(S)	>128(R)	>128(R)
Amino alcohol	Chloramphenicol	16(S)	8(S)	8(S)
Sulfonamide	Trimethoprim-sulfamethoxazole	8(R)	8(R)	8(R)
Cationic polypeptide	Polymyxin B	4(R)	4(R)	1(S)
	Colistin	8(R)	4(R)	1(S)

*MIC, minimum inhibitory concentration; R, resistant; I, intermediate; S, susceptible.^∗^In vitro antimicrobial susceptibility was performed by broth microdilution method and the MICs were interpreted according to Clinical and Laboratory Standards Institute (CLSI) criteria, except for tigecycline, colistin and polymyxin B, which interpretation were performed according to the EUCAST guidelines*.

### Conjugation Assay

To determine whether the colistin resistance was carried on a transferable plasmid, a conjugation experiment by filter mating assay (Smith and Guild, 1980) was performed with rifampicin-resistant *E. coli* C600 as the recipient strain. Overnight cultures of the original isolates and recipient *E. coli* C600 in LB broth were adjusted to a 0.5 McFarland standard. A 10 μl aliquot of each culture was individually added to 2 ml of fresh LB broth and then incubated at 37°C for 6 h. The original strains (20 μl) were then separately conjugated with *E. coli* C600 (60 μl) on a microporous membrane. Transconjugants were selected on MacConkey agar plates supplemented with colistin (2 μg/mL) and rifampicin (40 μg/mL), and putative transconjugants were confirmed by both PCR and an antimicrobial susceptibility test (above 22 antibiotics). The mobilization efficiency was calculated as the number of transconjugant colonies divided by the number of donor colonies (Wang et al., 2011).

### Multilocus Sequence Typing

The clonal lineage of the *E. coli* EC11 strain was studied using MLST. MLST was performed as previously described (Tartof et al., 2005). The seven conserved housekeeping genes (*adk, fum*C, *gyr*B, *icd, mdh, pur*A, and *rec*A) were chosen as targets^3^ and PCR fragments were sequenced. The alignments of these sequences were determined using DNAMAN software. These sequences were then analyzed using the facility provided by the above-mentioned online tool to assign allele numbers and define the ST and CC.

Furthermore, in order to explore possible genetic relationships between *E. coli* EC11 and other *E. coli* isolates harboring *mcr* reported worldwide, we performed a systematic review of the literature on *mcr* published in the NCBI-Pubmed database between November 2015 and March 2018. A phylogenetic tree was constructed using a NJ method by MEGA5.0 software, where the phylogenetic relationships among different strains were analyzed based on nucleotide differences. In addition, we conducted cluster analysis of these strains to understand the relationship between the different ST groups. The eBURST algorithm was used to group strains according to their allelic profiles by employing a user-specified group definition as well as drawing a rough sketch^4^ to show the genetic relationship.

### Whole Genome Sequencing

Genomic DNA of *E. coli* strain EC11 was extracted from an overnight culture using the TIANamp Bacteria DNA Kit (Tiangen Biotech Beijing Co., Ltd., China) according to manufacturer’s instructions. WGS data were generated using short-read (Illumina, San Diego, CA, United States), producing 2 × 251-bp paired-end reads, and long-read (Pacific Biosciences, Menlo Park, CA, United States) technology. The raw data were assembled using SPAdesv3.9.0 (Bankevich et al., 2012). Gene prediction and annotation were done with Glimmer 3.02 and BLAST. All sequences were deposited under the Bioproject PRJNA436212. Serotypes, plasmid replicons, and *E. coli* virulence genes were identified by using SerotypeFinder1.1, PlasmidFinder1.3, and VirulenceFinder1.5, respectively, available from the Center for Genomic Epidemiology^5^. Insertion sequence (IS) elements were identified using ISfinder^6^. Additional characterization of chromosomal resistance determinants was performed using the CARD Resistance Gene Identifier^7^, and ResFinder^8^ was used to detect acquired resistance genes commonly located on mobile genetic elements. The sequence comparison and map generation were performed using BLAST^9^ and Easyfig version 2.1 (Sullivan et al., 2011). Conjugal transfer components of the plasmids were performed using *oriT*finder (Li et al., 2018).

## Results

### Identification of *mcr-1*-Positive *E. coli* Isolates

In our study, out of 120 *E. coli* isolates collected from dairy cow fecal samples in May 2016 in Shanghai, only the *E. coli* isolate EC11 (Supplementary Figures S1, S2) carried the *mcr-1*gene, and none of these isolates carried *mcr-2/3* determinants or the allelic variants.

### Susceptibility to Antimicrobial and Conjugative Compounds

According to EUCAST standards, the resistance cutoff of *E. coli* to colistin is 2 mg/L and the *E. coli* EC11 strain exhibited the lower level of colistin resistance (8 μg/mL) (Table 1). *E. coli* EC11 also showed resistance to polymyxin B, and trimethoprim-sulfamethoxazole; but it was susceptible to other 19 common antibiotics, including amoxicillin-clavulanic, ampicillin, piperacillin, cefotaxime, ceftazidime, cefoxitin, cephazolin, cefepime, imipenem, meropenem, amikacin, gentamicin, kanamycin, tetracycline, tigecycline, ciprofloxacin, levofloxacin, nalidixic acid, chloramphenicol (Table 1). PCR results showed that *E. coli* EC11 didn’t carry the β-lactamase genes, including *bla*_TEM_, *bla*_SHV_, *bla*_CTX-M_, *bla*_KPC_, and *bla*_NDM_. Furthermore, the double disk test suggested that *E. coli* EC11 was a non-ESBL producing isolate (Supplementary Figure S3).

In addition, the filter mating assays indicated that the *mcr-1*-carrying plasmid could be successfully transferred from the donor (*E. coli* EC11) to the recipient (*E. coli* C600) with an average efficiency of 4.6 × 10^-5^. The MIC value of the transconjugant EC11-T to colistin was 8 μg/mL, which showed an eightfold increase when compared with the recipient *E. coli* C600 (1 μg/mL). The transconjugant *E. coli* EC11-T was also found to have resistance to nalidixic acid, trimethoprim-sulfamethoxazole and polymyxin B.

### A Diversity of the *mcr-1* Positive *E. coli* Isolates

Multilocus sequence typing (MLST) showed that *E. coli* EC11 belonged to the ST278 lineage. Based on the literature review, details of the *E. coli* strains harboring *mcr* genes, including the source and year of isolation, the presence of the MDR phenotype, ST, and allelic profile, are presented in Supplementary Table S2. A total of 245 STs were identified among the 616 *E. coli* isolates, indicating a high degree of genotypic diversity.

**FIGURE 1:**
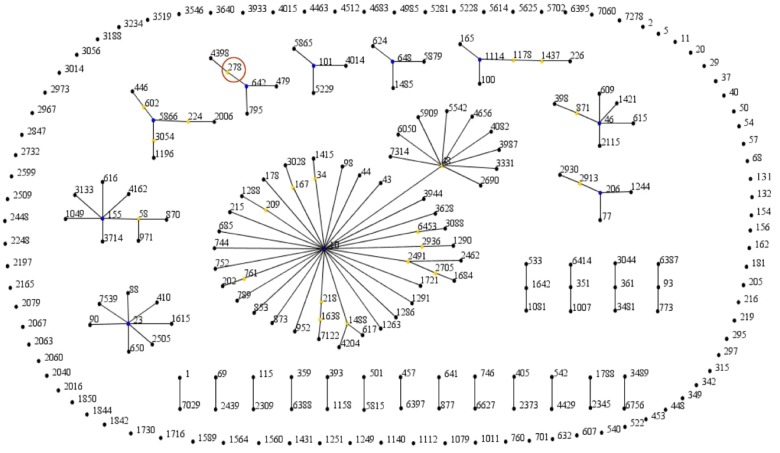
The eBURST cluster analysis of the genetic relationships of the *mcr*-positive *Escherichia coli* strains. The analysis is based on allelic profiles of MLST data and displays clusters of linked and individual unrelated STs. The digital represents the ST type, each black node represents a sequence type, the blue nodes represent clonal ancestors, the yellow nodes represent clonal subpopulation ancestors, and the red circle represents the sequence type of *E. coli* EC11 in this study.

The application of eBURST resolved the 245 STs into 10 clonal complexes (CC10, CC206, CC46, CC1114, CC648, CC101, CC642, CC6866, CC55, and CC23). CC10 remained the most populated clonal complex and ST10 was defined as the ancestral type of CC10 (Figure 1). The geographical distribution of the different STs is shown in Supplementary Table S3. These ST types were distributed in more than 35 cities across six continents. ST10 was isolated on five continents and China was the country where the most *mcr*-positive *E. coli* strains were found, with as many as 162 different STs being discovered.

**FIGURE 2:**
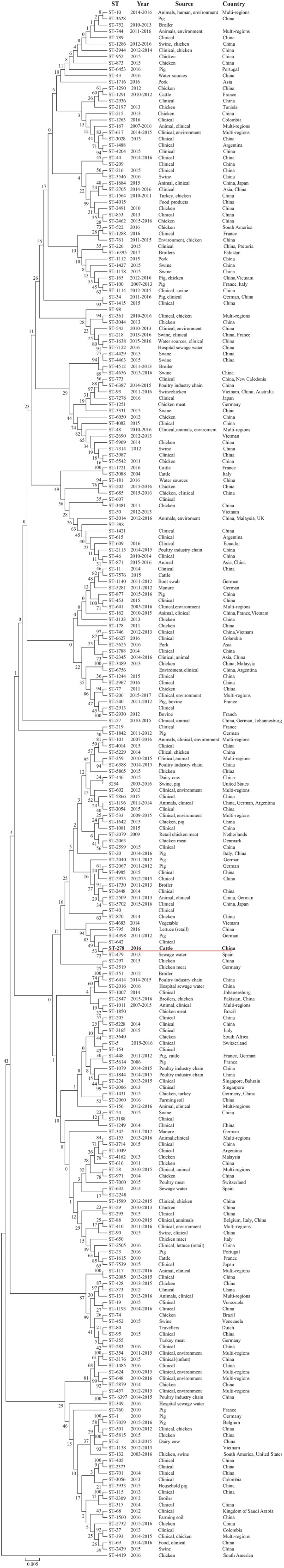
Neighbor-joining tree of 245 concatenated sequences of *E. coli* harboring *mcr-1* from multiple sources in different countries. The numbers at the nodes represent bootstrap values based on 500 replications. In bold and red underline presented the *E. coli* EC11isolate. ST, sequence type.

A NJ tree representing the concatenated sequences of the seven housekeeping gene fragments in 245 *mcr*-positive *E. coli* isolates of different ST types is shown in Figure 2. The phylogenetic analyses revealed that *E. coli* isolates harboring *mcr* genes were distributed in different lineages, and the isolated *E. coli* EC11 was located on a single branch rather than belonging to one of the ST10 branches.

### Genome Features of *E. coli* EC11 Harboring *mcr-1*

**FIGURE 3:**
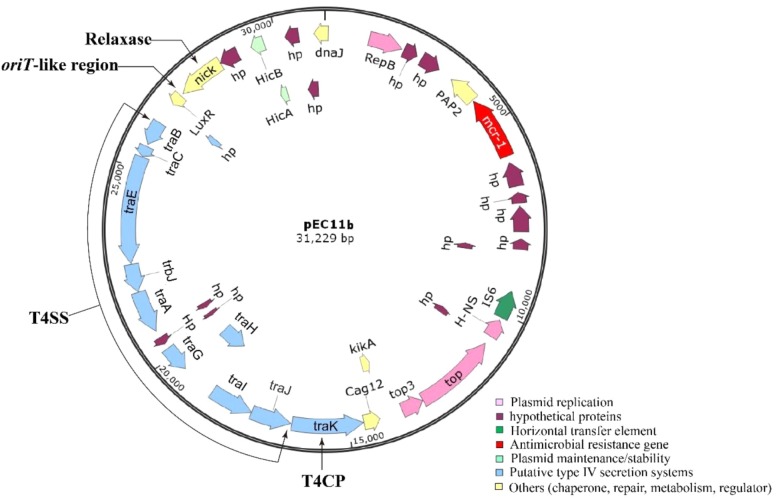
Map of the *mcr-1*-containing plasmid pEC11b isolated. The *mcr-1* gene is marked in red. Figure was created with by the software SnapGene Viewer.

Whole gene sequencing (WGS) revealed that the serotype of the *E. coli* EC11 strain was H7. *E. coli* EC11 consisted of a chromosome and four circular plasmids (pEC11a, pEC11b, pEC11c, and pEC11d) (Table 2). The chromosome genome size presented 4,933,784 bp, with a G+C content of 47.6%. With an exception of the *mcr-1*, unexpectedly, any other resistance genes were not defective in EC11. WGS results revealed the *mcr-1* gene, which showed 100% BLASTn identities to the known *mcr-1* gene of the reference plasmid pHNSHP45 of *E. coli* SHP45 (Liu et al., 2016). The *mcr-1* gene was only located on plasmid pEC11b, which was 31,229 bp in length and had an average G+C content of 41.40%, encoding 38 ORFs (Figure 3). Using PlasmidFinder, the plasmid pEC11b had a typical IncX4 plasmid backbone encoding replication, conjugation apparatus and stability functions, and was probably responsible for the movement of the plasmid between different bacterial hosts. The type II toxin–antitoxin module *hicA/hicB* was also identified in pEC11b. The putative virulence genes, such as *gad* (coding for glutamate decarboxylase), *lpf*A (long polar fimbriae) and *iss* (increased serum survival siderophore), were found in the chromosome of *E. coli* EC11.

**Table 2:** General features of *E. coli* EC11 genomes.

Replicons	Accession number	Size(bp)	MLST	Plasmid typing	Antibiotic resistance	GC (%)	ORF numbers	tRNA genes	rRNA genes
Chromosome	CP027255	4,933,784	ST278	–	–	50.77	4,648	85	22
pEC11a	CP027256	103,336	–	IncFIB	–	48.08	119	0	0
pEC11b	CP027257	31,229	–	IncX4	*mcr-1*	41.74	38	0	0
pEC11c	CP027258	31,467	–	–	–	48.41	46	0	0
pEC11d	CP027259	6,812	–	ColRNAI	–	47.69	9	0	0

### Genome Features of *mcr-1*-Carried Plasmid

**FIGURE 4:**
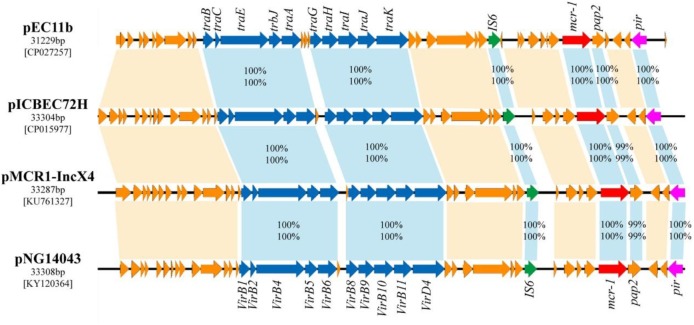
Linear comparison of complete plasmid sequences of plasmid pEC11b from *E. coli* EC11 (this study, accession no. CP027257), pICBEC72H from *E. coli* ICBEC72H (CP015977), pMCR1-IncX4 from *Klebsiella pneumoniae* SZ04 (KU761327) and pNG14043 from *Salmonella* NG14043 (KY120364). The arrows represent the position and transcriptional direction of the ORFs. Genes associated with the type IV secretion systems are indicated by blue arrows, resistance genes are indicated by red arrows, replication initiation protein are indicated pink, while accessory genes are indicated by jacinth arrows. Insertion sequences are highlighted in green arrows. The percentages of amino acid identity (above) and nucleic acid similarity (below) are shown between the homologous genes.

BLASTn analysis showed that the backbone of the plasmid pEC11b (GenBank accession number CP027257.1) was strikingly similar with (the query cover of 100% and the identities 99%) other previously sequenced *mcr-1-*carrying IncX4 plasmids, such as pICBEC72H of *E. coli* (isolated in Brazil; the GenBank accession no. CP015977.1), pMCR1-IncX4 of *Klebsiella pneumoniae* (China; KU761327.1), and pNG14043 of *Salmonella* (China; KY120364) (Figure 4). In all, these IncX4 plasmids bearing *mcr-1* showed very high architectural conservation.

An approximately 2.6 kb *mcr-1-pap2* element was identified in the above-mentioned plasmids pEC11b, PICBEC72H, pMCR1-IncX4, and PNG14043. In addition, an IS*6* element was identified in pEC11b, IS*26* was identified in PICBEC72H and PNG14043, and *tnpA* was identified in pMCR1-IncX4 (Figure 4). The promoter sequences of *mcr-1* in all the aforementioned sequences were similar to that of pAf23 and pAf48 reported by Poirel et al (Poirel et al., 2016) as well as pMCR1_IncI2 and BJ10 by Zhang et al (Zhang et al., 2017) (Supplementary Figure S4).

The putative conjugal transfer components of pEC11b were also detected by using *oriT*finder. A *tra* gene cluster encoding a T4SS belonging to Type P was predicted on pEC11b. It encoded a relaxase (*C6C13_26300*) belonging to the MOB_P_ family. It also encoded a T4CP (*C6C13_26225*) belonging to the VirD4 subfamily. The *oriT*-like region (coordinate: 27,146-27,223 bp) contained a pair of 14-bp IRs (GCAGGTGAGCAAAG…CTTTGTTCACCTGC). This evidence confirms that the plasmid pEC11b is a conjugative plasmid.

## Discussion

Colistin has been widely used as a veterinary drug for the treatment of enterobacterial infections and as an in-feed additive to promote healthy development in food-producing animals, especially in swine and poultry production (Kempf et al., 2013, 2016). Transfer of colistin resistance among bacteria in the gastrointestinal tract of livestock animals is a probable route for the dissemination of these bacteria (Fernandes et al., 2016b; Guenther et al., 2017). These routes can be via the food chain or direct human contact with animals as well as through contamination of fresh and seawater systems (Zhang et al., 2016; Zurfuh et al., 2016). In addition, the persistence of *mcr-1* in the human gastrointestinal tract microflora provides another route for dissemination of these bacteria (Chen et al., 2017). In this study, the *mcr-1-*carrying plasmid could be conjugated into *E. coli* C600 isolates *in vitro*. The *mcr-1* gene, if present in gut microbiota, can therefore be horizontally transmitted between different species in the microbiota.

Self-transmissible IncX4-type plasmids are now accepted as key vehicles responsible for the dissemination of the *mcr-1* gene among Enterobacteriaceae worldwide (Fernandes et al., 2016a; Sun J. et al., 2017; Wang et al., 2017). In this study, we identified an IncX4-type plasmid carrying *mcr-1* in *E. coli* EC11, pEC11b, which was nearly identical to the other IncX4 plasmids bearing *mcr-1* in GenBank. IncX4 plasmid architecture is highly conserved and studies have shown similar IncX4 plasmids bearing *mcr-1* from different species. These species were isolated from different geographic locations and belonged to different STs (Sun J. et al., 2017; Wang et al., 2017). Plasmid pEC11b has four typical conjugal modules: an origin of transfer (*oriT*-like) region, a T4CP gene, a relaxase gene, and a gene cluster for the bacterial T4SS apparatus. The T4SS can act as a conjugative machine in conjugative plasmids (Cascales and Christie, 2003). These gene clusters are vital to the HGT of intra- and inter-species bacterial resistance genes (Frost et al., 2005). Also, the plasmid pEC11b contains the *mcr-1*-*pap2* cassette which has proven that it could be horizontally transferred into diverse plasmid replicon types (Li et al., 2016).

Multilocus sequence typing (MLST) is a powerful genetic fingerprinting technique for molecular epidemiology and population genetic studies of bacterial pathogens (Maiden et al., 1998; Urwin and Maiden, 2003; Maiden, 2006). In this study, we reported the first recorded instance of an *mcr-1* producing *E. coli* EC11 belonging to the ST278 lineage. We performed a MLST-based analysis of the *mcr*-positive *E. coli* population structure among 616 isolates collected in different laboratories in over 35 countries since 2016. The 245 STs among the 616 isolates indicate that the *mcr*-positive *E. coli* population is extremely diverse. Applying eBURST and NJ tree analyses simultaneously in this global dataset allows for better resolution in discerning the epidemiology and genetic population structure of *mcr*-positive isolates. Combined with previous studies (Matamoros et al., 2017), we speculate that the diversity in ST types of these *E. coli* strains may be related to highly promiscuous plasmids disseminating *mcr* genes. It also indicates that *mcr-1* has a huge risk of vertical transmission and may become more widespread and prevalent in the future. A ST which is highly disseminated in food, environment, animals, and human intestinal samples is ST10 (Matamoros et al., 2017; Sun P. et al., 2017). The epidemic clone ST131 (Ortiz de la Tabla et al., 2017), ST648 (Yang et al., 2016), and ST206 (Zheng et al., 2018) were reported to be the most common STs associated with various β-lactamases, including ESBLs, NDM, and KPCs, etc. Many reports indicated that bacteria carrying *mcr-1* were often associated with ESBLs (Sun et al., 2016). In this study, *E. coli* EC11 only conferred resistance to polymyxin B, colistin, and trimethoprim-sulfamethoxazole, which are antibiotics that are extensively prescribed in veterinary medicine (Catry et al., 2015).

Currently, a number of countries have already restricted the use of colistin in animal production. China has now stopped the use of colistin as an antibiotic growth promoter (Walsh and Wu, 2016). South Africa has responded to the threat of losing colistin as an antibiotic for human health through a program to advance national stewardship of colistin across the ‘One Health’ platform (Mendelson et al., 2018). The discovery of the *E. coli* strain EC11 with resistance to colistin in Shanghai emphasizes the importance of vigilance in detecting new threats like *mcr* genes to public health.

## Conclusion

In this work, we report the first case of colistin-resistant *mcr-1* gene in *E. coli* strain EC11 isolated from dairy cow feces in Shanghai, China. We show that this *E. coli* strain carrying the *mcr-1* gene can transfer resistance through HGT. This study confirms the need to monitor and survey the use of colistin and other types of antibiotics to enable proactive and effective strategies (e.g., risk assessment and risk management) for preserving the efficacy of antibiotics in the future.

### Nucleotide Sequence Accession Number

The genome sequences of the chromosome and four plasmids of the *E. coli* strain EC11 were deposited as GenBank accession no. CP027255-CP027259.

## Author Contributions

YZ, YP, and HL conceived and supervised the study. FB designed the experiments. FB and ZZ performed the experiments. FB and XL analyzed the data. BN and XL revised the paper. PM edited the paper. FB wrote the paper.

## Conflict of Interest Statement

The authors declare that the research was conducted in the absence of any commercial or financial relationships that could be construed as a potential conflict of interest.

## Footnotes

1 https://clsi.org/

2 http://www.eucast.org/

3 http://mlst.warwick.ac.uk/mlst/dbs/Ecoli

4 http://eburst.mlst.net/v3/enter_data/single/

5 http://genomicepidemiology.org/

6 https://www-is.biotoul.fr/search.php

7 https://card.mcmaster.ca/analyze/rgi

8 https://cge.cbs.dtu.dk/services/ResFinder/

9 http://blast.ncbi.nlm.nih.gov

## Abbreviations

CC: clonal complexes
CLSI: Clinical and Laboratory Standards Institute
CRE: carbapenem-resistant Enterobacteriaceae
*E. coli*: *Escherichia coli*
ESBL: extended spectrum β-lactamase
EUCAST: European Committee on Antimicrobial Susceptibility Testing
HGT: horizontal gene transfer
IRs: inverted repeats
IS: insertion sequences
MDR: multidrug-resistant
MIC: Minimum Inhibitory Concentration
MLST: Multilocus Sequence Typing
NJ: Neighbor-joining
ORFs: open reading frames
PCR: polymerase chain reaction
PEA: phosphoethanolamine
SEM: scanning electron microscope
ST: sequence type
T4CP: type IV coupling protein
T4SS: type IV secretion system
TEM: transmission electron microscope
WGS: whole-genome sequencing
XDR: extensively drug-resistant
