# Supplementary material for: A mcr-1-Carrying Conjugative IncX4 Plasmid in Colistin-Resistant Escherichia coli ST278 Strain Isolated From Dairy Cow Feces in Shanghai, China
Source: Front Microbiol. 2018 Nov 30;9:2833. doi: 10.3389/fmicb.2018.02833 (PMC6287198; doi:10.3389/fmicb.2018.02833)
Supplement: Supplementary file 1 [file Data_Sheet_1.PDF]

A *mcr-1*-carrying conjugative IncX4 plasmid in colistin-resistant *Escherichia coli* ST278 strain isolated from dairy cow feces in Shanghai, China

Fengjia Bai[1†], Xiaobin Li[5†], Ben Niu[1], Zhaohuan Zhang[1], Pradeep K. Malakar[1]

Haiquan Liu[1,2,3,4], Yingjie Pan[1,2,3], Yong Zhao[1, 2, 3*]

[1] *College of Food Science and Technology, Shanghai Ocean University, Shanghai 201306*
[2] *Shanghai Engineering Research Center of Aquatic-Product Processing & Preservation, Shanghai 201306*
[3] *Laboratory of Quality & Safety Risk Assessment for Aquatic Product on Storage and Preservation (Shanghai), Ministry of Agriculture, Shanghai 201306*
[4] *Engineering Research Center of Food Thermal-processing Technology, Shanghai Ocean University, Shanghai 201306*
[5] *State Key Laboratory of Microbial Metabolism, Joint International Research Laboratory of Metabolic & Developmental Sciences, School of Life Sciences & Biotechnology, Shanghai Jiao Tong University, Shanghai 200030, China*
† These authors have contributed equally to this work.

* **Correspondence**: Yong Zhao, College of Food Science & Technology, Shanghai Ocean University, Shanghai, 201306, China. yzhao@shou.edu.cn

SUPPLEMENTARY DATA

Table S1 | Primers used in this study.
Table S2 | A total of 245 unique sequence types (STs) of *mcr*-positive *E. coli* strains were summarized from previous literatures.
Table S3 | Geographic distribution of the 616 *mcr-1*-positive *Escherichia coli* isolates.
Figure S1 | Scanning electron microscopy (A) and transmission electron microscope (B) images of *E. coli* EC11.
Figure S2 | (A) PCR identified the *E. coli* EC11. (B) PCR-based determination of presence of *mcr-1* gene in the *E. coli* EC11. M, DNA marker; kb, kilo-base pair.
Figure S3 | Double disk test the *E. coli* EC11.
Figure S4 | Analyses of the *mcr-1* promoter. Prokaryotic promoter analysis by the Neutral Network Program of Promoter Prediction (http://www.fruitfly.org/seq_tools/

promoter.html). Designations: S, transcription start site; M, methionine and translation initiation site; RBS, ribosome binding site. The *mcr-1* promoter sequences are indicated with the corresponding -10 and -35 boxes being underlined according to Poirel L's and Zhang's work.

Table S1 Primers used in this study

Primer	Nucleotide sequence (5’→3’)	Fragment size (bp)	Annealing temperature (℃)	References
16SrRNA	F: AGAGTTTGATCCTGGCTCAG<br>R: GGTTACCTTGTTACGACTT	1465	60	This study
mcr-1	F：CGGTCAGTCCGTTTGTTC<br>R：CTTGGTCGGTCTGTAGGG	309	52.5	(Liu et al., 2016a)
mcr-1	F: GCGGATGAATGCGGTGCG<br>R: ATGATGCAGCATACTTCTGTG	1623	55	(Liu et al., 2016b)
mcr-2	F: AATCGTTGCACTTGGTTTGACA<br>R: ATAACAAACCCCACCCCACC	1914	60	(Xavier et al., 2016)
mcr-3	F: TCGTTGCACTTGGTTTGACA<br>R: CAAATGGTCGCAACCAGCAA	2152	60	(Yin et al., 2017)
bla_{SHV}	F: GAGCGAAAGATCCACTATCG<br>R: GGTATCCCGCAGATAAATCA	525	55	(Li et al., 2018)
bla_{TEM}	F: ATGAGTATTCAACATTTCCGTG<br>R: TTACCAATGCTTAATCAGTGAG	847	54	(Li et al., 2018)
bla_{CTX-M}	F: CAGGAGTTTGAGATGATGAG<br>R: GAGCGCTCCACATTTTTTAG	910	55	(Li et al., 2018)
bla_{KPC}	F: GCTACACCTAGCTCCACCTTC<br>R: TGGAGGGCCAATAGATGATT	945	55	(Li et al., 2018)
bla_{NDM}	F: CAGGCAACAGCCGAACGA<br>R: CGTTAGATTGGCTTACACCATTAGA	1193	54	(Li et al., 2018)

Table S3 | Geographic distribution of the 616 *mcr*-positive *Escherichia coli* isolates.

Continents	Country	No. of strains	No. of STs	Mainly STs
Asia	China	378	162	2,10,46,48,88,101,117,167,744, etc.
	Japan	4	4	1684,5702,7278,7539
	Thailand	1	1	167
	Bangladesh	1	1	48
	Singapore	2	2	224,2006
	Pakistan	5	5	10,155,361,2847,6395
	Vietnam	15	15	10,48,165,410,457,476,1011, etc.
	Malaysia	6	6	117,410,744,3014,3489,4162, etc.
	Kingdom of Saudi Arabia	1	1	68
	United Arab Emirates	1	1	131
	Bahrain	2	2	224,648
	Other countries	8	8	117,206,354,871,1716,2345,2705,5625
Europe	France	27	17	10,100,101,744,1291,1721, etc.
	Italy	13	10	10,88,100,101,650,744,1011, etc.
	Spain	9	4	10,167,479,632
	Austria	1	1	10
	Norway	1	1	10
	Germany	32	24	10,34,156,410,1011,1196,2509, etc.
	Portugal	7	7	10,23,38,101,156,744,6453, etc.
	Belgium	4	4	10,88,167,7029
	Denmark	5	5	48,131,359,744,2063
	Switzerland	5	5	5,48,117,359,7060
	Dutch	3	3	80,744,1011
	Netherlands	4	3	117,359,2079
	UK	1	1	3014
	Other countries	4	3	10,58,167
North America	United States	6	6	57,117,457,132,410,3234
	Canada	1	1	624
South America	Colombia	8	8	10,37,101,744,1263,3056,6627
	Brazil	5	5	74,101,167,354,1850
	Argentina	10	10	101,410,602,615,641,744,1049, etc.
	Venezuela	2	2	19,452
	Ecuador	1	1	609
	Other countries	5	5	10,48,132,522,4419
Africa	Pretoria	2	2	10,226
	South Africa	1	1	3640
	Johannesburg	5	5	57,101,624,1007
	Tunisia	1	1	2197
Oceania	Australia	2	2	93,167
	New Caledonia	2	2	617,773
Unknown	—	25	21	10,40,57,98,349, etc.

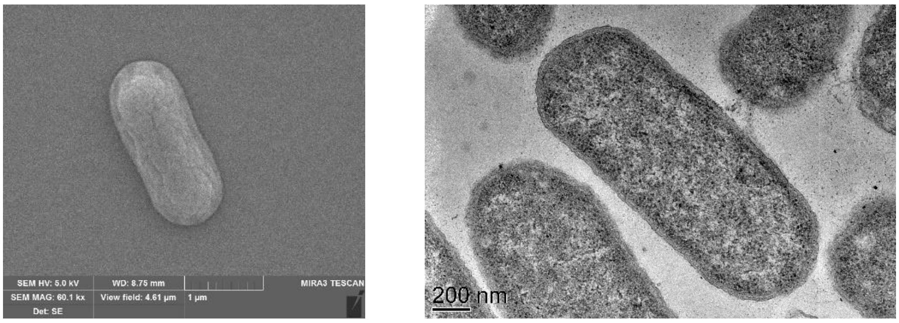

(A) **(B)**

Figure S1 | Scanning electron microscopy (A) and transmission electron microscope (B) images of *E. coli* EC11

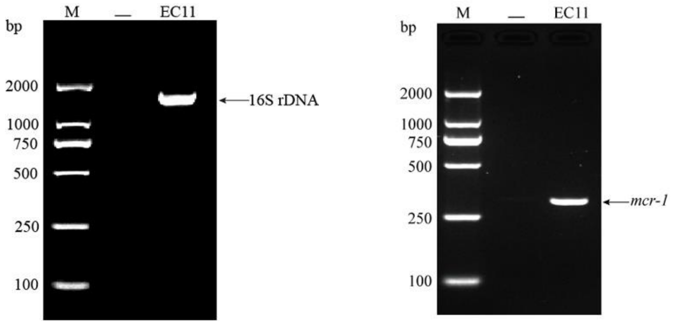

(A) **(B)**

Figure S2 | (A) PCR identified the *E. coli* EC11. (B) PCR-based determination of presence of *mcr-1* gene in the isolated *E. coli* EC11. M, DNA marker; kb, kilo-base pair.

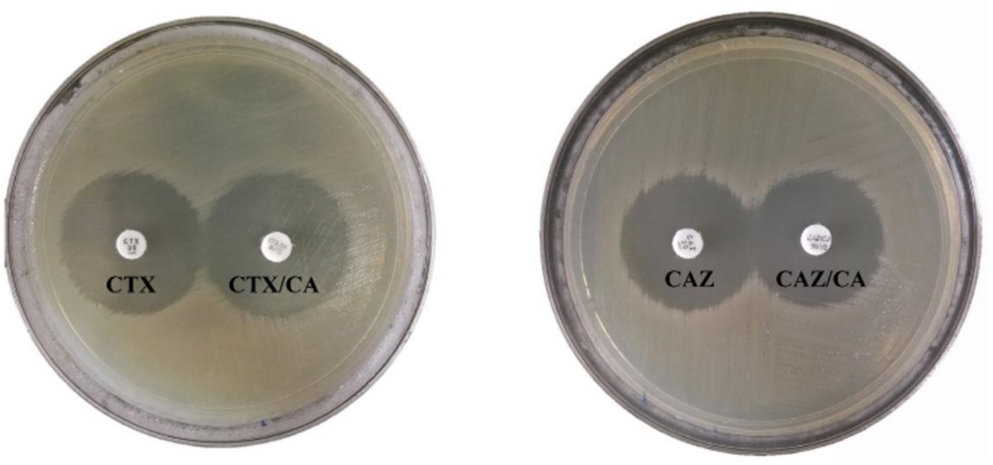

(A) **(B)**

Figure S3 | Double disk test the isolate of *E. coli* EC11. **(A)** CTX/CA (30µg/10µg): 30mm, CTX (30µg): 30mm; **(B)** CAZ/CA (30µg/10µg): 28 mm, CAZ (30µg): 28mm.

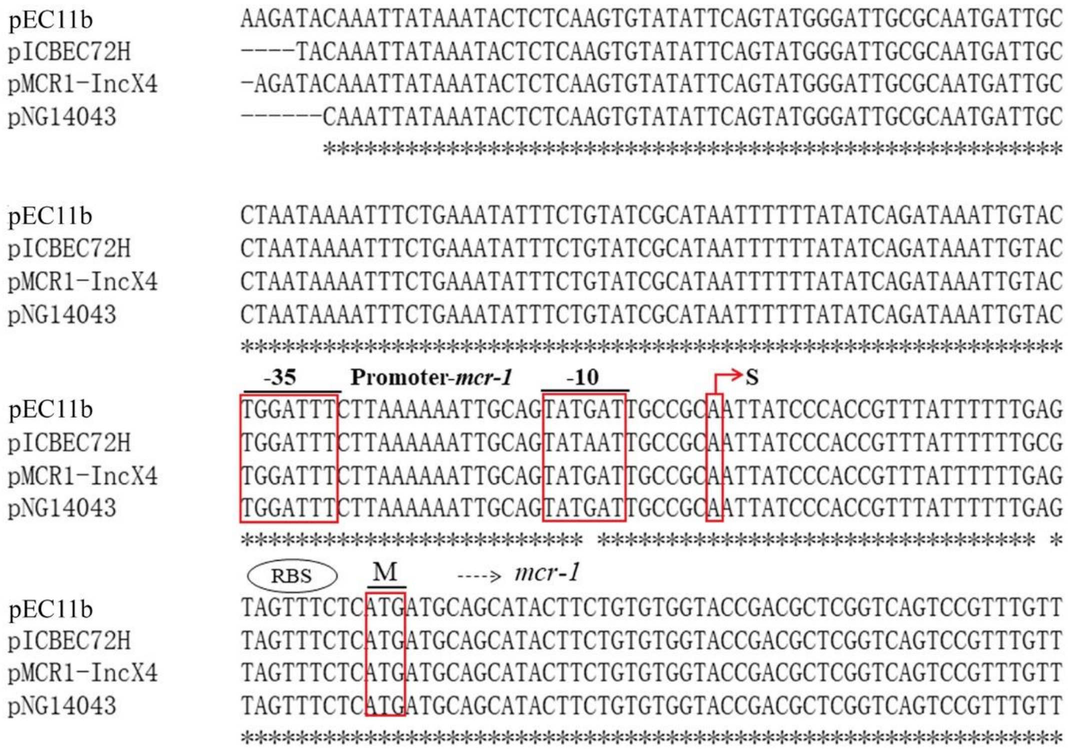

FIGURE S4 | Analyses of *mcr-1* promoter. Prokaryotic promoter analysis by the Neutral Network Program of Promoter Prediction (http://www.fruitfly.org/seq_tools/ promoter.html). Designations: S, transcription start site; M, methionine and translation initiation site; RBS, ribosome binding site. The *mcr-1* promoter sequences are indicated with the corresponding -10 and -35 boxes being underlined according to Poirel L's and Zhang's work.

Reference

Li, Q., Wang, H., Xu, Y., Bai, X., Wang, J., Zhang, Z., et al. (2018). Multidrug-Resistant *Escherichia albertii*: Co-occurrence of beta-Lactamase and MCR-1 Encoding Genes. *Frontiers in Microbiology* 9. doi: 10.3389/fmicb.2018.00258.

Liu, Y.Y., Wang, Y., Walsh, T.R., Yi, L.X., Zhang, R., Spencer, J., et al. (2016a). Emergence of plasmid-mediated colistin resistance mechanism MCR-1 in animals and human beings in China: a microbiological and molecular biological study. *Lancet Infect Dis* 16(2)**,** 161-168. doi: 10.1016/S1473-3099(15)00424-7.

Xavier, B.B., Lammens, C., Ruhal, R., Kumar-Singh, S., Butaye, P., Goossens, H., et al. (2016). Identification of a novel plasmid-mediated colistin-resistance gene, *mcr-2*, in *Escherichia coli*, Belgium, June 2016. *Euro Surveill* 21(27). doi: 10.2807/1560-7917.ES.2016.21.27.30280.

Yin, W., Li, H., Shen, Y., Liu, Z., Wang, S., Shen, Z., et al. (2017). Novel Plasmid-Mediated Colistin Resistance Gene *mcr-3* in *Escherichia coli*. *MBio* 8(3). doi: 10.1128/mBio.00543-17.
